# Prolonged Radiation-Induced Gastritis Following Yttrium-90 Radioembolization: A Case of Persistent Dyspepsia and Endoscopic Surveillance

**DOI:** 10.7759/cureus.102508

**Published:** 2026-01-28

**Authors:** Muhammad Haris Latif, Abdelrahman Gadallah, Ayesha Kang, Hani El-Halawani, Sandeep Sen

**Affiliations:** 1 Internal Medicine, SSM Health St. Mary's Hospital, Saint Louis, USA; 2 Gastroenterology, SSM Health Depaul Hospital, Saint Louis, USA; 3 Oncology, SSM Health St. Mary's Hospital, Saint Louis, USA

**Keywords:** gastrointestinal complications, intrahepatic cholangiocarcinoma, intravascular microspheres, non-target embolization, radiation-induced gastritis, transarterial radioembolization, vonoprazan, yttrium-90 radioembolization

## Abstract

Yttrium-90 (Y-90) transarterial radioembolization (TARE) is an established therapy for unresectable hepatic malignancies. Although generally safe, Y-90 therapy can occasionally lead to extrahepatic complications, including gastrointestinal injury. Here, we present a case of delayed-onset Y-90-induced gastritis in a 72-year-old woman with intrahepatic cholangiocarcinoma. Following Y-90 radioembolization, the patient developed delayed gastrointestinal symptoms. Subsequent endoscopy revealed erythematous gastric mucosa with erosions, and biopsy showed intravascular microspheres consistent with radiation injury. Her symptoms improved with vonoprazan therapy and supportive management. This case thus illustrates delayed gastrointestinal toxicity as a rare complication of hepatic radioembolization.

## Introduction

Yttrium-90 (Y-90) microsphere transarterial radioembolization (TARE) is an established therapy for unresectable primary and metastatic hepatic malignancies, delivering targeted beta-radiation to tumor-bearing hepatic segments while preserving adjacent parenchyma [1]. Although TARE generally demonstrates a favorable safety profile, complications may arise if microspheres are inadvertently deposited in non-target vascular territories, such as the gastrointestinal tract [2]. These complications may manifest as radiation-induced gastritis, ulceration, or duodenitis, with reported incidence rates ranging from 3% to 11% depending on the study [3-5]. Identified risk factors include procedural misplacement of microspheres and pre-existing gastrointestinal conditions, highlighting the necessity of meticulous procedural planning and vigilant patient monitoring. Preventive strategies, including angiographic mapping and coil embolization, are essential for minimizing the risk of non-target embolization [6]. These measures facilitate precise microsphere delivery and decrease the likelihood of serious complications.

Although Y-90-induced gastritis typically develops within weeks following therapy, rare biopsy-confirmed cases may present several months after radioembolization. This delayed onset, even after an unremarkable initial follow-up, emphasizes the necessity for sustained clinical vigilance [7,8]. Diagnosis and management of delayed Y-90-induced gastritis require a comprehensive approach, including endoscopy with biopsy to confirm radiation-induced changes. Histopathological findings, including mucosal inflammation, necrosis, and vascular endothelial damage, characterize these changes. Imaging studies further delineate the extent of gastrointestinal involvement [9]. Management generally involves proton pump inhibitors (PPIs) to reduce gastric acid secretion and promote mucosal healing, supplemented by additional symptomatic treatments as needed. Ongoing patient monitoring and prompt intervention are crucial to prevent complications and optimize clinical outcomes.

## Case presentation

**Figure 1:**
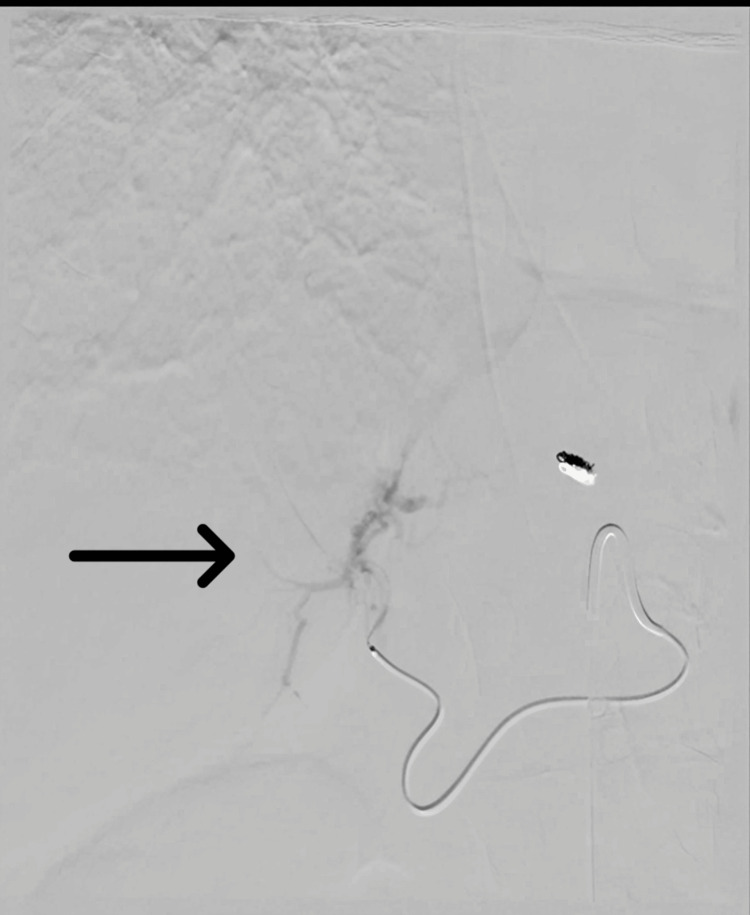
Selective hepatic angiogram Selective hepatic angiography showing targeted administration of yttrium-90 microspheres into the segment 4 branch of the left hepatic artery (black arrowhead). The catheter tip is positioned within the segment 4 arterial branch to deliver therapy to the left hepatic lobe while minimizing non-target arterial flow.

A 72-year-old Caucasian woman with advanced hepatic and lymphatic malignancy underwent Y-90 radioembolization targeting the left hepatic lobe and segment four on December 11, 2023. Before this intervention, she had received multiple systemic therapies, including chemotherapy/immunotherapy (cisplatin, gemcitabine, and durvalumab) and an isocitrate dehydrogenase 1 (IDH1) inhibitor (ivosidenib), without disease control. Pre-procedural hepatic arterial mapping and selective vessel embolization were performed on December 8, 2023. The embolization technique used microcoils and embolic particles, targeting the left hepatic artery to reduce non-target deposition. A total dose of 68.2 millicuries (mCi) of Y-90 was administered (Figure 1). The procedure was uneventful.

**Figure 2:**
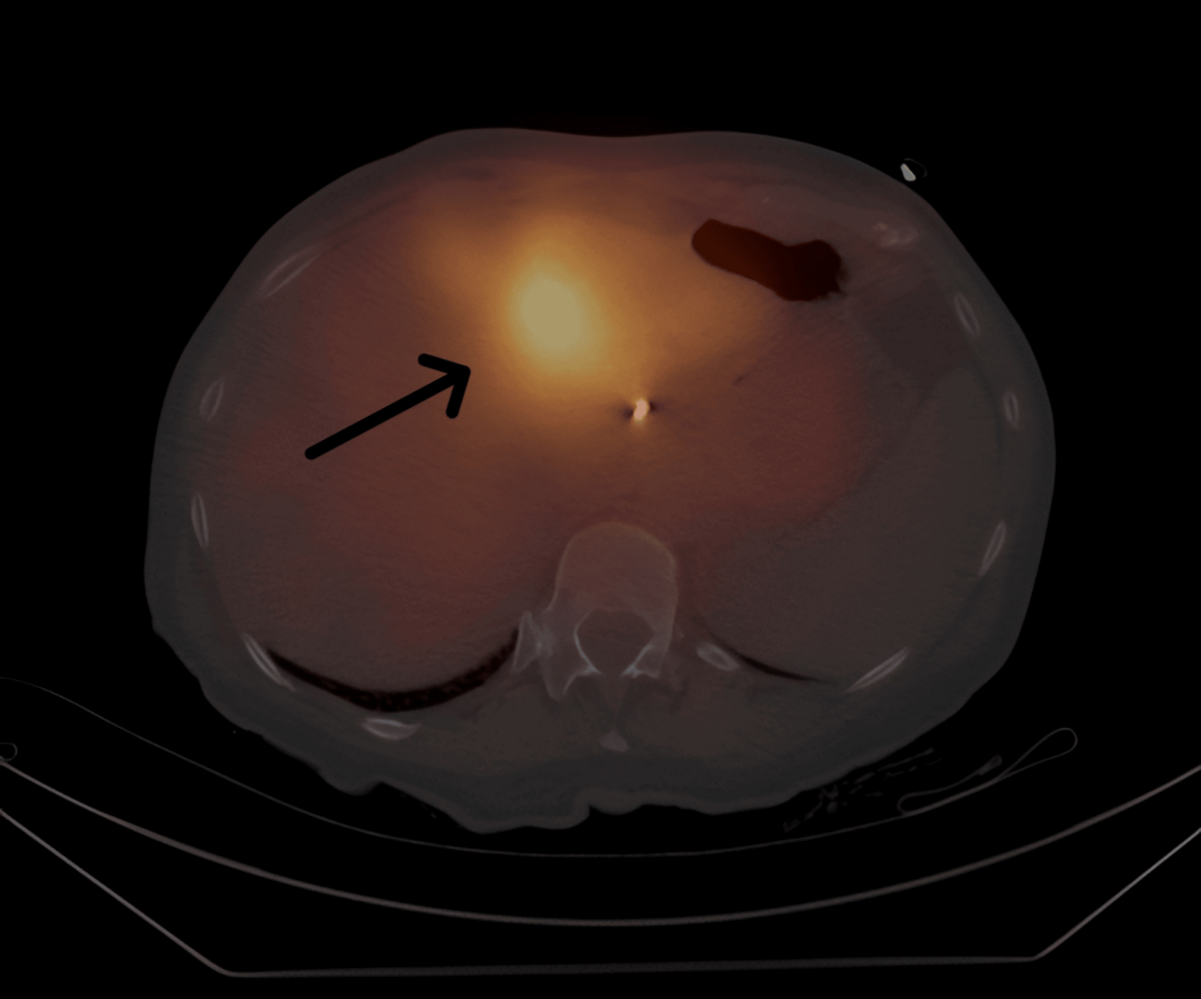
A post-therapy yttrium-90 (Y-90) Bremsstrahlung single-photon emission computed tomography (SPECT) scan. Post-treatment Bremsstrahlung SPECT imaging demonstrating tracer distribution localized to the left hepatic lobe without significant extrahepatic uptake, confirming appropriate yttrium-90 microsphere delivery (black arrowhead).

Approximately four weeks after the Y-90 procedure, the patient developed worsening indigestion and upper stomach pain but no vomiting, blood in vomit, or black stools. She did not use nonsteroidal anti-inflammatory drugs or drink alcohol. Labs showed mild increases in liver enzymes, with other values normal (Table 1). Single-photon emission computed tomography/computed tomography (SPECT/CT) imaging demonstrated high sensitivity in confirming that the radiotracer remained in the left liver, with no extrahepatic spread (Figure 2).

**Table 1:** Laboratory findings Reference ranges are shown for comparison. WBC = white blood cell; ALT = alanine aminotransferase; AST = aspartate aminotransferase; ALP = alkaline phosphatase; INR = international normalized ratio

Parameter	Patient Value	Reference Range	Units
Hemoglobin	12.8	12.0–16.0	g/dL
WBC	7.2	4.0–10.0	×10⁹/L
Platelets	220	150–400	×10⁹/L
AST	68	0–40	U/L
ALT	74	0–41	U/L
ALP	120	30–120	U/L
Total Bilirubin	1.3	0.3–1.2	mg/dL
Albumin	3.4	3.5–5.0	g/dL
INR	1.1	0.8–1.2	–

**Figure 3:**
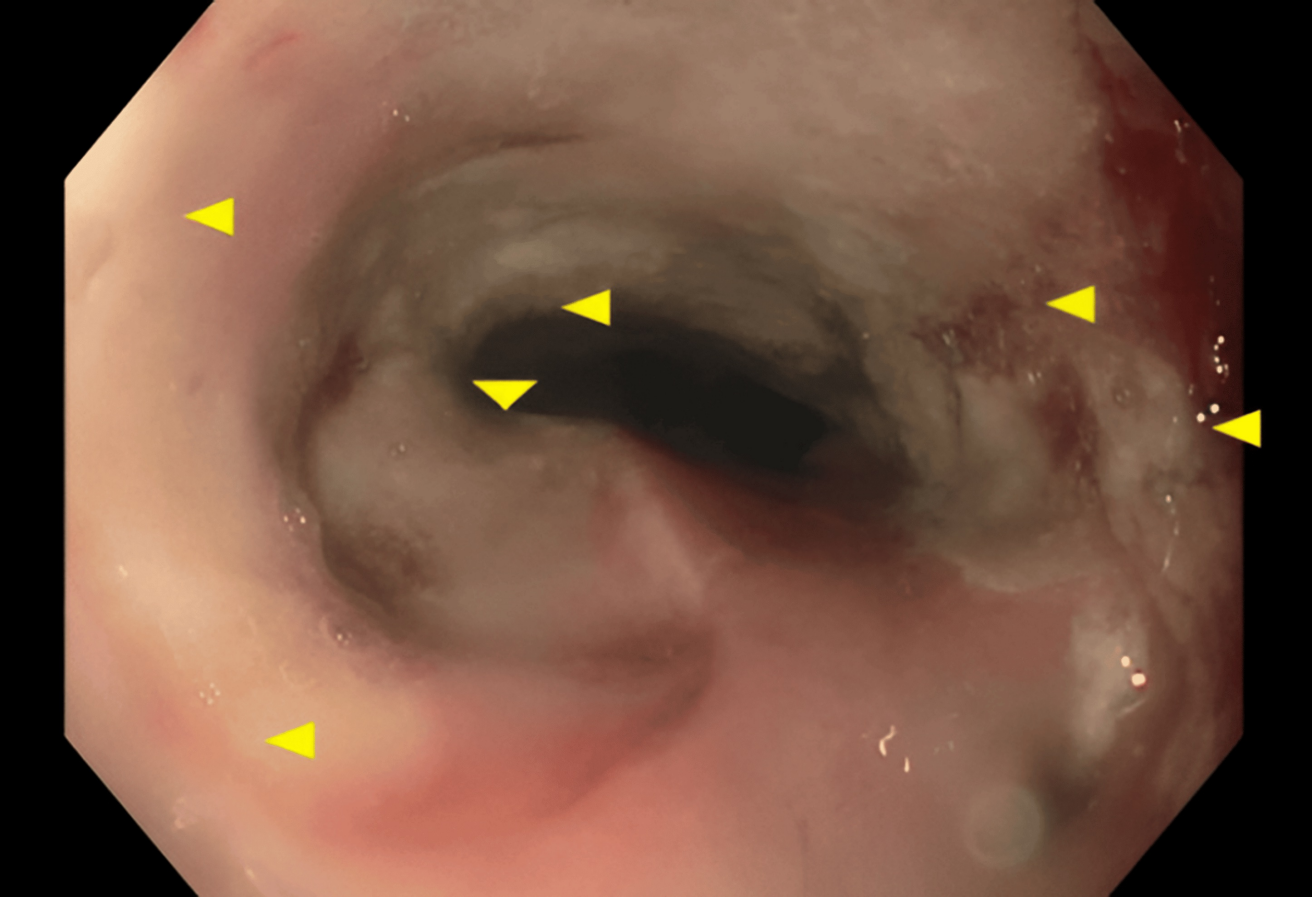
Endoscopic findings of upper gastrointestinal mucosal injury Endoscopic image demonstrating erythematous gastric mucosa with patchy erosions (yellow arrows mark regions of inflammation).

Given the new symptoms, an initial esophagogastroduodenoscopy (EGD) showed red, irritated stomach lining with patchy erosions and a benign distal esophageal stricture (Figure 3). Biopsies showed healing tissue and abnormal glands, suggesting early radiation damage. Histopathological examination revealed key features characteristic of radiation-induced injury patterns, including vascular changes such as intimal thickening and luminal narrowing, as well as focal necrosis. Later stomach biopsies showed a thin stomach lining in the lower part, with moderate short- and long-term inflammation and cellular changes from irritation. The ulcer showed moderate-to-severe inflammation and reactive cell changes, but no *Helicobacter pylori *was detected. Biopsies from the esophagus found eroded lining with severe inflammation and pus-like debris, but no fungi. These results fit with gastritis caused by Y-90 radiation therapy.

Despite these findings, initial treatment brought only some relief. Esophageal dilation, started two weeks after the Y-90 procedure, was scheduled every four weeks and provided temporary improvement. Acid suppression therapy began concurrently with the first dilation session. However, indigestion persisted, necessitating four additional EGDs over the subsequent two months. Repeated endoscopies showed no new ulcers or malignancy, though the lining remained red and fragile.

**Figure 4:**
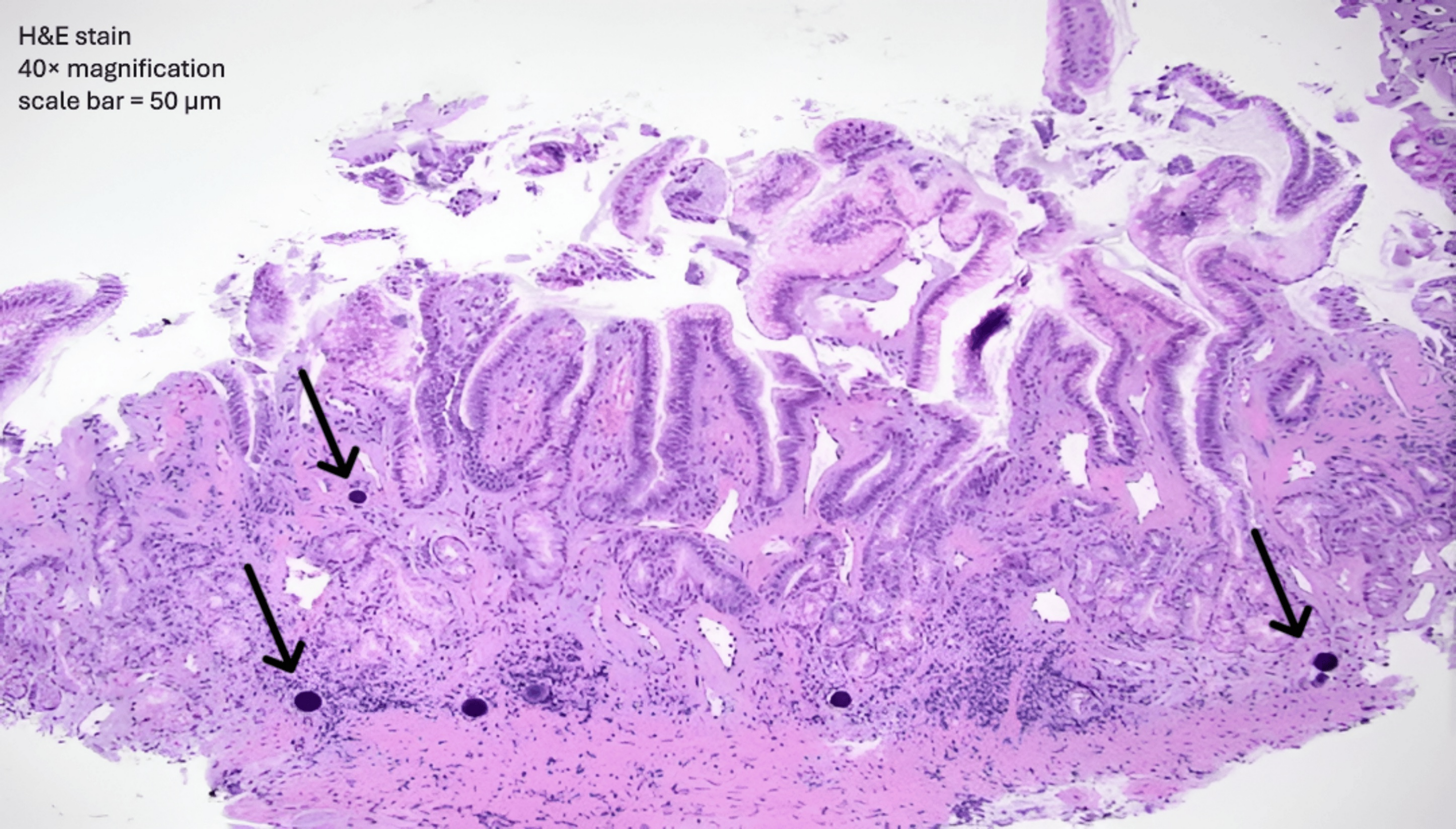
Gastric biopsy with intravascular yttrium-90 microspheres. Hematoxylin and eosin (H&E)–stained gastric biopsy showing intravascular yttrium-90 microspheres (arrowhead) within small submucosal vessels, accompanied by chronic inflammatory infiltrate and mucosal injury. Magnification 40×; scale bar = 50 µm.

**Figure 5:**
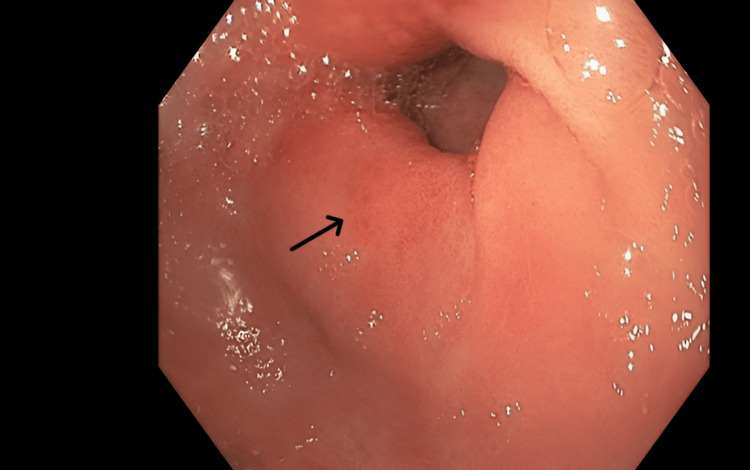
Follow-up endoscopic evaluation Gastric antrum showing healed mucosa with resolution of erythema.

At the 12-month follow-up, further investigation revealed intravascular Y-90 microspheres in gastric biopsy specimens (Figure 4), confirming radiation-induced vascular injury due to non-target microsphere deposition. Notably, no dysplasia, malignancy, or *Helicobacter pylori* infection was detected in any sample. Following this confirmation, the patient was initiated on a daily potassium-competitive acid blocker (P-CABs) and advised dietary modifications. Over the following year, she underwent serial endoscopic dilations for esophageal stricture, with gradual symptomatic improvement. Dyspepsia resolved, and subsequent endoscopies demonstrated stable mucosal healing without recurrent ulceration or bleeding (Figure 5). The duodenum remained normal throughout all assessments.

## Discussion

Y-90 TARE is a well-established treatment for liver tumors that cannot be removed surgically. Still, if microspheres escape the liver, they can injure the upper gastrointestinal tract. The rate of severe gastroduodenal ulcers varies widely, from about 0% to 29%, but is usually under 5% at experienced centers. These differences are due to variations in technique, embolization approach, and the type or amount of microspheres used. This case, characterized by persistent dyspepsia, endoscopic gastritis, and histologic evidence of intravascular microspheres, exemplifies a rare but recognized complication of Y-90 radioembolization [5].

The leading causes of this complication are non-target embolization through hepaticoenteric collaterals and reflux during infusion. Studies have found that arterial stasis during injection is a major independent risk factor. This highlights the importance of careful flow management and close monitoring of hemodynamic changes during the procedure. Ulcers usually start on the serosal surface, suggesting vascular or wall injury rather than acid-related mucosal disease [6].

Symptoms typically appear between 10 days and five months after TARE; however, delayed onset beyond this period, such as in this patient, remains unusual. These case reports describe both early (within about two weeks) and late presentations. In this case, symptoms started about four weeks ago, which is typical. However, finding intravascular beads on histology at around 12 months is unusual and provides valuable clinical insight [7].

Initial biopsies often show acute-on-chronic gastritis with reactive epithelial changes and signs of ischemia or regeneration. Still, they may not reveal microspheres, especially if they are confined to small areas or to deeper submucosal vessels. A definite diagnosis requires seeing black or purple microspheres within blood vessels, which may only be present in later or deeper samples. Ogawa et al. described a range of findings, including ulceration, granulation tissue, epithelial cell death, and loss of mucin, with beads measuring about 40 µm found in small vessels [8]. Similarly, Castro et al. found Y-90-blocked vessels in biopsies of pyloric ulcers and noted that endoscopic findings may be unclear without the proper clinical context [4].

Imaging after the procedure is essential to assess where the microspheres have gone and to detect any activity outside the liver. Traditionally, bremsstrahlung SPECT has been used, but studies show that Y-90 PET/CT, especially time-of-flight PET with resolution recovery, gives better image quality and detection. This helps identify small amounts of extrahepatic accumulation. When available, PET/CT enables earlier detection, especially when endoscopic findings are unclear [10].

There is no single proven treatment for this complication. Most reports suggest using acid suppression with PPIs or P-CABs, such as vonoprazan, along with mucosal protection, dietary changes, and targeted endoscopic treatment for bleeding. Strictures may need repeated balloon dilation, as in this patient. Steroids have sometimes been used for severe inflammation. For cases that do not respond or are complicated by bleeding or blockage, surgery such as diversion or gastrectomy may be needed. In this case, the patient had sustained symptom control with P-CAB and repeated endoscopic dilation, without new ulcers or bleeding, which aligns with conservative, organ-preserving approaches in the literature [1].

Before treatment, angiographic mapping with coil embolization of at-risk hepaticoenteric branches and careful catheter placement are standard. Still, small, unnoticed collateral vessels or changes in blood flow can allow microspheres to reach non-target areas. Current guidelines stress thorough pre-planning, including technetium-99m macroaggregated albumin (99mTc-MAA) mapping, dosimetry, and post-treatment checks, with Y-90 PET now preferred when possible. Routine acid suppression is commonly used for prevention, though its effectiveness is not proven.

## Conclusions

Y-90 radioembolization is a widely utilized therapy for liver cancer; however, inadvertent microsphere deposition can lead to delayed gastric injury. This case report describes a patient who developed Y-90-induced gastritis several months after treatment, with diagnosis confirmed by identifying microspheres in gastric biopsy specimens. Persistent dyspepsia may serve as an indicator of radiation-induced injury, highlighting the need for prompt evaluation and conservative management. In this instance, treatment with a P-CABs and scheduled endoscopic surveillance effectively controlled symptoms and promoted mucosal healing. Careful vascular mapping and diligent post-procedural monitoring are critical for preventing and detecting these rare complications.
